# Stressful life events and tinnitus: Reflections and speculations

**DOI:** 10.1007/s00106-024-01501-3

**Published:** 2024-08-02

**Authors:** Laurence McKenna, Florian Vogt

**Affiliations:** 1 Ear Institute, University College London, 332 Gray’s Inn Road, London, UK; 2 Royal National ENT and Eastman Dental Hospitals, University College London, London, UK

**Keywords:** Stress, Critical life events, Psychological impact, Emotional regulation, Stress response, Stress, Kritische Lebensereignisse, Psychologische Auswirkungen, Emotionale Regulierung, Stressreaktion

## Abstract

The role of life events has not been extensively studied in the context of tinnitus. There is, however, evidence about the psychological impact of tinnitus and about the influence of psychological processes as mediators of the tinnitus experience. On the basis of this evidence, the possibility that stressful life events can act as a trigger for tinnitus is discussed; although the evidence is fragmentary and indirect, the possibility cannot be discounted. It is argued that the onset of tinnitus and the associated clinical encounters can constitute an acute stressful life event, and the ongoing experience of tinnitus can be regarded as a chronic stressful life event. Interactions between ongoing tinnitus and other life events are discussed. Possible mechanisms in terms of stress influencing predictive processing and signal detection judgments are considered.

## Abstract (de)

Die Rolle von Lebensereignissen wurde im Kontext von Tinnitus nur wenig untersucht. Es gibt jedoch Hinweise auf die psychologischen Auswirkungen von Tinnitus und auf den Einfluss psychologischer Prozesse als Mediatoren des Erlebens von Tinnitus. Unter Berücksichtigung dieser Hinweise wird die Möglichkeit diskutiert, dass stressige Lebensereignisse Auslöser für Tinnitus sein können; obwohl die Belege fragmentarisch und indirekt sind, kann diese Möglichkeit nicht ausgeschlossen werden. Es wird argumentiert, dass der Beginn des Tinnitus und die damit verbundenen klinischen Kontakte ein akutes belastendes Lebensereignis darstellen können und die fortlaufende Tinnituserfahrung als chronisches belastendes Lebensereignis betrachtet werden kann. Wechselwirkungen zwischen fortbestehendem Tinnitus und anderen Lebensereignissen werden diskutiert. Mögliche Mechanismen im Hinblick auf Stress, der die prädiktive Verarbeitung und Beurteilungen der Signalerkennungbeeinflusst, werden in Betracht gezogen.

The etiology of tinnitus has been thoroughly studied, with common causes identified as noise-induced hearing loss, presbycusis, Meniere’s disease, infections, and neurological conditions such as whiplash injury and acoustic neuroma [1]. Alongside these diverse biological origins, the notion that stressful life events are related to tinnitus is widely accepted. Perhaps this is unsurprising because of the often paradoxical nature of tinnitus. While the majority of people with tinnitus have some degree of hearing loss, some people with apparently normal hearing experience tinnitus while others with significant hearing loss do not [2]. There are numerous aspects to the relationship between stressful life events and tinnitus, few of which are well researched.

## The nature of stress

A challenge in any discussion of stressful life events is to define “stress” [3] because it can refer to an event or a response. McEwen describes a stressful life event as one interpreted as threatening, triggering physiological and behavioral responses [4]. The role of interpretation is also integral to cognitive behavior therapy (CBT; [5]). There are, however, some conventions about what sort of event is likely to be “stressful.” Events like the death of a loved one or a terminal illness diagnosis are widely seen as stressful. It is likely that an event that is directly harmful will be considered stressful. Alternatively, events may be interpreted as stressful if they demand adaptation, particularly if that requires more resources than are available to the person or interferes with the pursuit of important goals. These factors overlap and it is not clear whether any one of them is necessary or sufficient for the generation of stress. These possibilities also suggest that the impact of stress is likely to be cumulative.

McEwan [4] explains stress responses through hypothalamic–pituitary–adrenal (HPA) and autonomic nervous system (ANS) activity, noting that “allostatic load” indicates chronic stress due to excessive or ineffective allostasis (p. 174). Behaviorally, stress responses include fight-or-flight reactions, vigilance, anxiety, and behavioral changes.

There is a need to distinguish the impact of acute stress from that of chronic stressors. Persistent adversity and long-term threats are more closely related to poor function than acute life events are, and it is likely that acute life events have more profound effects by changing long-term stressors and resources [3, 6]. It is possible that such stresses play a role in precipitating acute events, e.g., long-term financial problems leading to homelessness, overworking leading to relationship breakdown. Research into the effects of stressful life events therefore needs careful design. To date, this research has not taken place in the field of tinnitus. It has therefore been necessary to consider the effects of stressful life events from what is more generally known about the patient suffering from tinnitus.

## Stressful life events as a cause of tinnitus onset

It is recognized that, although most people exposed to stressful life events do not get sick, the experience of such events can influence the onset or progression of many illnesses [3]. Bessel Van Der Klok [7] makes a persuasive argument linking trauma, and other life stresses, to a number of health problems; unfortunately, he does not address the issue of tinnitus. The suggestion that stress triggers tinnitus, however, fits well with the wider discourse linking stress and illness.

The idea that stress might induce tinnitus is an old one. It can be found in the literature from antiquity to the present day. The Ancient Greek poet Sappho (630–570 B.C.) describes the experience of being lovestruck as provoking a range of physical symptoms including *ears buzzing* [8]. J. K. Rowling portrays Harry Potter as experiencing tinnitus in a number of situations including when experiencing strong emotions [9]. There are numerous other examples in popular literature [10]. The Tinnitus UK online article “All About Tinnitus” [11] clearly assumes that there is evidence to support the idea tinnitus can be triggered by stress. It states: “It [tinnitus] can be started by a stressful live event. When the event is over, the tinnitus may stop.” It can be recognized that the descriptions in the literature seem to be referring to transient tinnitus, a tinnitus that appears in response to non-enduring stressful lived life events.

There are, however, also reports of stress being linked to non-transient tinnitus. In several studies, patients report that tinnitus onset is directly linked to stress. Hazell [12] reported that in 75 of 100 consecutive patients, the onset of tinnitus was associated with a psychological or social trigger. He noted that in no case was there any evidence of change in audiometric status over the time that tinnitus developed. Mazurek et al. [13] cite unpublished data from Hébert pointing to the link between stress and tinnitus onset. They state that 53.6% of patients reported that tinnitus onset occurred during a period of stress and almost all named a specific event such as divorce, job loss, or bereavement, suggesting that “exposure to stress triggered tinnitus.” Handscomb’s qualitative investigation [14] of patients who had therapy for tinnitus distress [15] revealed that patients believed their tinnitus was triggered by stress. One patient, Suzannah, believed that a series of stressful experiences, mostly related to her work, had caused her tinnitus to start. She stated:

“There’s been a number of traumatic things that I’ve had to deal with in these last three years … and that feels like that’s the start” (p. 174).

In some studies, tinnitus patients were asked if they had a psychiatric disorder prior to tinnitus, which may be an indication of stress causing tinnitus. For example, in a group of tinnitus patients, it was recorded that among those who met the criteria for a psychiatric disorder, the disorder preceded tinnitus in 64% of cases [16]; it should be noted that 62% of the sample also had inner ear dysfunction. The presence of psychological problems prior to the onset of tinnitus was also observed by others [17].

Other studies demonstrate an association between posttraumatic stress disorder (PTSD), psychological distress closely linked to life events, and the presence of tinnitus. Hinton et al. [18] noted a high prevalence of PTSD and tinnitus among a group of Cambodian refugees in the United States compared with the normal population. Those with PTSD were also more likely to have tinnitus and PTSD was more severe in those who also had tinnitus. Data from US military veterans suggest a similar picture, with studies showing strong positive associations between PTSD and tinnitus [19, 20]. In line with Hinton et al. [18], Fagelson [19] reported that patients affected by both tinnitus and PTSD had more severe PTSD and greater tinnitus handicap than those who had tinnitus without PTSD.

The present authors have a combined clinical experience of almost 50 years. The impression gained from this is that tinnitus onset happens in the context of stressful life events in approximately 50% of cases. The present authors, however, as psychologists, see a subset of tinnitus patients. The patient whose tinnitus began when she was grieving for her recently deceased husband, or whose tinnitus started when faced with financial problems, or during an emotionally painful divorce or the patient who developed tinnitus when his teenage son went to prison for drug offences and worsened when the “boy” returned from jail as a difficult “man” are all likely to be familiar to clinicians. A history of tinnitus onset following a stressful life event or during a period of high stress is a recurrent theme in clinical practice.

These studies and reports are suggestive of a causal link between stressful life events and tinnitus, but caution is needed. Alternative explanations are clearly possible. Stressful life events and psychiatric disorders are common in the population and some of those afflicted will also develop tinnitus. It may also be that pre-existing psychological difficulties lead a person to seek help for tinnitus. Furthermore, PTSD populations are often exposed to more conventional tinnitus triggers, such as loud sounds [18, 21], and by definition having trauma and tinnitus is a greater burden than developing tinnitus by “natural” causes. The role of psychological trauma in triggering tinnitus therefore remains complex, if persuasive.

## Tinnitus onset as a stressful life event

It seems likely that, once triggered, the onset of tinnitus itself acts as a stressful life event. There is evidence that many tinnitus patients are dissatisfied by their early encounters with GPs and tinnitus clinicians [22]. Patients are frequently told some version of, “this is tinnitus, there is no cure, you must learn to live with it.” In one study, 80% of participants reported receiving this message [23]. When patients are already distressed by tinnitus, this message can compound their distress because they hear that they must learn to live with feeling as bad as they do at that time. Handscomb [14] quotes one patient’s reaction to being told he had tinnitus:

“Tinnitus will affect my mental health … Life will never be the same again” (Mick) pp. 167–168.

“It was annoying but then … I thought I’d had a nervous breakdown, an acute stress response …” (Mick) p. 173.

Another participant said:

“Very quickly I had suicidal thoughts because I thought, ‘How can I possibly live with this?’” (Jessica) p. 168.

Many tinnitus patients believe health professionals underestimate the impact of tinnitus and this lack of validation exacerbates their distress [24, 25]. Patients described the early experience of tinnitus in terms of its disruptive impact but also in terms of the stressful impact of healthcare encounters [26]. This included a sense of abandonment by health professionals who said they could not help and referred them to others. Patients described feeling stressed by having the burden of managing tinnitus placed on them. They described a troubling uncertainty about understanding information about tinnitus, multiple appointments, waiting for results, seeking coping strategies, and adjusting daily life and their self-identity. The consequences of such early encounters can be far reaching [27], as negative thinking shortly after the onset of tinnitus predicts a poor outcome [28]. It seems therefore that contact with some clinicians results in iatrogenic damage that turns a clinical consultation into a stressful life event. Positive interactions with tinnitus clinicians, however, can reduce anxiety [29].

## Chronic tinnitus as a stressful life event

Setting tinnitus onset aside, the chronic experience of tinnitus seems to constitute a negative life event for many, and the louder the tinnitus, the worse the problem [30]. There is a high prevalence of emotional distress in tinnitus patients [13, 15, 31], with the majority of patients meeting criteria for a formal psychiatric diagnosis [16, 32–34]. It has even been suggested that tinnitus distress itself represents a psychiatric condition [35], although this suggestion is perhaps only an attempt to categorize tinnitus. Apart from reduced well-being, the presence of tinnitus is associated with reduced cognitive functioning, in particular attention [36]. Patients with tinnitus certainly make negative appraisals about tinnitus, including about its unnaturalness, it escalating, it interfering with normal activity, an inability to cope, and possible psychiatric consequences [37] – and even about spiritual disaster [18]. There seems to be a consensus opinion that the experience of tinnitus is a stressful process.

The stress experienced from tinnitus, with its cognitive and behavioral correlates, may in turn further worsen the experience of tinnitus. Cognitive models of tinnitus distress [15, 28] argue that the way in which people think about and behave in response to tinnitus can lead to alterations in tinnitus perception, preoccupation, and distress. This idea is clearly recognized by many patients. The point is made by one of Handscomb’s [14] study participants:

“I do ruminate a lot and it [tinnitus] seems to grow when I’m doing that” (Graham) p. 172.

The suggestion that psychological factors contributor to the ongoing awareness, loudness, and severity of tinnitus has been made by many researchers [38–40]. It has been found that tinnitus loudness and tinnitus distress are mediated by tinnitus acceptance, depression, and anxiety [41]. The iterative nature of the process implies that the psychological distress experienced is not only a reaction to tinnitus but acts as a “provocateur” of the experience. In this context it is important to note that the cognitive behavioral models describe the ongoing stress reaction not just in terms of a failure to habituate but also as potentially creating a greater sensitization to tinnitus.

## Stressful life events causing fluctuations in chronic tinnitus

The models of tinnitus distress discussed above focus on a stress reaction to tinnitus maintaining or exacerbating tinnitus awareness. They say little about the role of other life events in the experience of tinnitus. Technically, however, they leave open the possibility that other stressful events could add to the overall burden and through that route increase distress from tinnitus. While the cognitive model of tinnitus distress [15] is described as helpful by patients, some comment that it neglects the role of other stresses impacting on tinnitus [14].

“When I am stressed it is much more obvious, but I am learning to recognise that and actually see it as indicating to me what my state of mind is.” (Jessica) p. 175

Another patient reports:

“The sort of injustice and upset over some of the things that have happened in the last few years … is what I focus on … even though it then exacerbates and makes [tinnitus] worse.” (Suzannah) p. 172.

Surveys seem to corroborate this notion, showing that people who perceived the COVID-19 lockdown as generally stressful with increased grief, frustration, and stress reported a worsening of tinnitus distress [42]. Fagelson [20] noted that participants reported that tinnitus loudness was exacerbated during periods of stress. Empirical evidence also comes from Mazurek et al. [13] citing unpublished data from Hebert, noting that 52.8% of patients reported that tinnitus increased during stressful periods. It has also been found that higher levels of emotional exhaustion can be a better predictor of tinnitus distress than factors such as hearing loss [43]. An experimental study showed that healthy military personnel, with normal hearing, were more likely to report transitory tinnitus after shooting practice if their mood was worse [44]. The authors hypothesized that mood disturbance could make the ear more vulnerable to the impact of loud noise.

It is possible that a life history that contains significant stressful events predisposes a person to experience greater distress in response to tinnitus. Handscomb [14] noted that her participants raised this issue: “Several of the patients felt that their tinnitus was made worse by a pre-existing mental illness … all of them felt that these conditions intensified the emotional distress they felt in relation to their tinnitus” (p. 174). Using psychoanalytic concepts, it has been argued that tinnitus may disrupt the strategies that a person developed in response to historic stressors and as a consequence make the experience of tinnitus more impactful [45]. Those researchers noted that some participants presented a life narrative with ongoing struggles beyond tinnitus, which they were unable to resolve, suggesting that tinnitus makes it more difficult to cope with existing stresses. Studies linking PTSD and tinnitus support the contention that chronic stresses influence the tinnitus experience. It has been reported that tinnitus could provoke recall of traumatic events, flashbacks, nightmares, and exaggerated startle responses to unexpected sounds [19, 20].

In keeping with the theme of tinnitus acting as a reminder of other traumas, Dauman and Erlandsson [46] described a complex relationship between tinnitus and a previously non-disclosed trauma, which occurred 50 years earlier in the patient’s life. They argue that even when the memory of a trauma is unconscious, the feelings associated with it can be triggered by a similar experience, as, for example, in the form of a tone, a voice, or a place [45, 46]. Clinical experience suggests that there is something substantial in these arguments, although memories of the stressful events may also be conscious. For example, one of the present authors’ patients described the anxiety he experiences in response to tinnitus as feeling the same as he experienced as a child when severely reprimanded. The evocation of the earlier stress adds an extra burden to his experience of tinnitus.

The psychoanalytic ideas of Erlandsson and colleagues [45, 46] find similar expression in CBT theory. It is suggested that early life lessons lead to the development of *core beliefs* [5] that in turn help shape *intermediate beliefs* including a*ssumptions* about the world and *rules *for living. A negative core belief such as “I am not good enough” may lead a person to develop an assumption such as “If I show weakness with life’s challenges people will reject me” and to impose a rule such as “I must be strong at all times.” Conforming to such rules requires effort. A person may struggle to maintain that effort while also coping with tinnitus. This type of narrative occurs frequently in clinical practice and is an example of the way that historic stresses can influence the impact of tinnitus. These processes are perhaps indirectly reflected in the work linking anxious personality types with greater tinnitus distress [47].

The neurophysiological model of tinnitus [48] emphasizes the importance of other stressful life events in creating tinnitus distress. The theory suggests that tinnitus is an intrinsically neutral stimulus that can become classically conditioned to distress provoked by coincidental stressful life events. Hazel’s [12] observation that the onset of tinnitus is preceded by stressful life events in 75% of cases is seen as important evidence in support of the theory. This aspect of the theory, however, lacks clarity. To be consistent with classic conditioning principles, the process must be one whereby tinnitus precedes the recall of the coincidental stressful event, rather than tinnitus being triggered by the stressful event. Hazell’s [12] account therefore sits awkwardly with the conditioning aspect of the model. The processes in that model are more in keeping with the recall of trauma described in the PTSD studies [19, 20] and by Dauman and Erlandsson [46]. Habituation is a central concept in the neurophysiological model and with the psychological conceptualization of tinnitus suggested by Hallam et al. [49]. The latter authors suggested that the rules of habituation to tinnitus are the same as those governing habituation to other stimuli. Part of that understanding is that high levels of stress arousal will slow the process, thereby allowing for the possibility of other stressful life events influencing the experience of tinnitus.

The presence of life stressors is associated with poorer treatment outcome in other health domains and can predict outcome more than the severity of the health problem at the start of treatment [3]. Although patients’ accounts [14] imply this effect, there remains little empirical evidence about this possibility in the context of tinnitus. For example, it is not known whether people who continue to experience tinnitus distress in the long term are also experiencing other chronic stresses.

The literature, together with clinical experience, does suggest that the presence of other stresses influences the experience of tinnitus and that tinnitus may make it harder to cope with other stresses. The examples of an interaction between tinnitus and long-term stresses referred to in the tinnitus literature and those described by patients reflect the acute/chronic stress links described in the life events literature [6]. More empirical evidence is needed, but the idea that the “seed” of tinnitus can fall into “emotionally fertile soil” certainly seems credible. None of these interactions are meant to discredit clinical observations in which tinnitus exacerbation is attributed to other reasons or indeed is described as idiopathic.

## Mechanisms driving the impact of stress on tinnitus

Research indicates that stressful life events can influence the onset or progression of illnesses by affecting emotional regulation, behavior, and neurohormonal systems [3]. Cohen et al. suggest that exposure to stress may impact any disease that involves emotional regulation, health behaviors, hormonal activity, or the autonomic nervous system. Tinnitus fits this description. The distinctions between changes in emotional regulation, behavior, and neurohormonal activity, however, can be unclear, as these processes are likely interdependent.

A number of physiological processes involved in stress arousal are recognized as having an impact on the auditory system, and it has been argued that these may account for the provocative impact that stress has on tinnitus [13, 50]. More psychological processes, focusing on changes in information processing, add to the overall picture. Stress is a response to perceived threat. There is a survival benefit in making more “cautious” judgments when threatened; a false-positive judgment represents a less costly error than a false-negative one. Stress makes individuals more likely to perceive and report potential threats, even if those threats might not be present or are ambiguous. This is because stress arousal lowers the threshold for detecting these threats, leading to a bias in perception [51].

Sedley et al. propose a model of tinnitus based on predictive coding [52]. They suggest that spontaneous activity in the subcortical auditory pathway constitutes a “tinnitus precursor” that is normally ignored and taken to represent “silence.” They further suggest that certain triggers may alter the intensity or precision of this activity so that it is perceived as tinnitus. The authors posit that changes in attention can increase the precision of the precursor activity and helps maintain tinnitus awareness. This is in keeping with the processes of stress arousal leading to selective attention and distorted perception posited in the cognitive model of tinnitus distress [15] and may account for the observations that stress can increase the awareness of existing tinnitus.

The possibility that stressful life events can trigger tinnitus onset in an otherwise healthy auditory pathway remains intriguing, as stress alone is usually not considered to cause disease in healthy people [3]. Nevertheless, it is acknowledged that this view is accepted because there are challenges to gathering the evidence [3]. Many tinnitus patients, however, identify the precise timing of tinnitus onset and some can confidently link that timing with the occurrence of a life event [14]. Within the terms of Sedley and colleagues’ model, one could speculate that the stress arousal, through top-down processing, acts as a trigger to changes in the intensity or precision of the spontaneous auditory signal leading a person to perceive tinnitus. Research into auditory hallucinations offers something of a parallel. It has been noted that psychological stress commonly precedes bouts of verbal hallucinations and this has been explained in terms of compromised predictive processing and error correction [51]. These authors reported that increasing stress led healthy participants to produce more false-positive judgments about the presence of auditory signals [51], i.e., stress increased the likelihood that they would perceive an auditory signal for which there was no external sound. The effect was more evident in more anxious individuals and more likely to occur when there was an expectation that a signal would be present. Similar processes are potentially possible within the Sedley et al. model of tinnitus [52].

The suggestion that a psychological process might be a proximate cause of tinnitus may invite discussion of mind/body or chicken/egg puzzles. A reflection on one’s own personal experience, however, can provide clear examples of how thinking alone can change bodily state, e.g. blushing, fatigue, sexual arousal, etc. Such changes in bodily experience due to thinking are common. There is no reason to suppose that tinnitus is not subject to them. This, however, does seem like another area for further research and further thought.

## Summary

The tinnitus referred to in poetry and literature is essentially an acute physical manifestation of stress, similar to a racing heart, tight muscles, or dry mouth. One might speculate that this experience of tinnitus happens to many people but passes quickly and is not reported to clinicians. For some, however, the symptom itself may constitute an acute stressful life event that the person finds difficult or impossible to disengage from. The publication by Tinnitus UK [11] suggests that if tinnitus is triggered by stressful life events, it may cease when the stress stops. The disappearance of tinnitus in this way is not obvious in a clinical setting, but this may be because the person is less likely to bring the matter to a clinician. Although this is speculative, there is an argument that clinicians should routinely enquire about recent and chronic stresses and their juxtaposition to the onset of tinnitus.

A distinction is made in the literature between the processes that underpin the onset of tinnitus from those involved in the maintenance of it as a problem. Onset has been conceptualized as a reflection of altered auditory activity, while it has been suggested that psychological processes, particularly stress-related processes, are central to maintenance [36]. The division lines between these processes may be difficult to define. It may be that psychological processes, particularly changes in “top-down” processes of detection and attention are involved at the very start of the tinnitus experience. The psychiatrist and neuroscientist McGilchrist argues that, “attention changes what kind of a thing comes into being for us: in that way it changes the world” (p. 28; [53]). Van Der Kolk [7] argues that the effects of psychological stress “are not necessarily different from—and overlap with the effects of—(*neurological*) lesions” (p. 42). The possibility that stressful life events can trigger tinnitus can be considered in this context.

Investigating tinnitus patients’ thoughts, Wilson and Henry [37] noted that the most commonly endorsed cognition was, “why me?” An answer might be, “because the function of the auditory pathway has deteriorated.” This might suffice even allowing for the fact that hearing loss is not always present, and an appeal is made to the impact of a process such as hidden hearing loss [54]. Even if this is seen as an answer, then there is always the question of whether stress played a part in bringing about the changes in the auditory pathway. Further, it does not necessarily answer the question, “why now?” This is an important question if, as Hazel [12] noted among his patients, there is no other evidence of change in audiometric status. Clinicians can have some confidence in answering this question by attributing the onset of tinnitus, or changes in existing tinnitus, to changes in stress. That confidence may be increased with further research, particularly some longitudinal studies of people considered at risk of tinnitus. Until then, for clinicians, the etiology of tinnitus—whether due to stress or to structural or biological changes—is secondary to the imperative of alleviating the patient’s stress response to the condition. The principal goal is to assist patients in managing their stress reactions; helping them to make attributions of their tinnitus that feel most acceptable may touch upon life events and build upon a person’s coping skills to deal with these. It is possible that psychotherapeutic approaches equip the patient with skills that help them address life stresses as well as tinnitus, and it might be speculated that some of their benefit is realized through that route; another area for research. It can be argued that CBT impacts only on a person’s coping strategy [55] and does not address the origin of tinnitus more fundamentally. If, however, stress is seen as part of a process that drives the experience of tinnitus and potentially even plays a role in triggering it, then perhaps CBT can be seen as having a more fundamental role. This should have implications for how tinnitus budgets are spent.

## Practical conclusion

- Care is needed when arriving at conclusions: Life events research has not been conducted in the field of tinnitus in the way it has in some other areas.
- It is certainly possible to emphasize the shortcomings in the literature and question the justification of these speculations and conclusions.
- A clinician looking at the evidence must guard against confirmation bias.
- A call for further research is, however, justified because clinical experience and the available evidence suggest that the links between tinnitus and stressful life events go beyond naïve realism.

## Declarations

For this article no studies with human participants or animals were performed by any of the authors. All studies mentioned were in accordance with the ethical standards indicated in each case.

## References

[1] Patil JD et al (2023) The association between stress, emotional states, and tinnitus: a mini-review. Front Aging Neurosci: 1131979 10.3389/fnagi.2023.1131979 PMC10188965 37207076

[2] Henry JA et al (2014) Underlying mechanisms of tinnitus: review and clinical implications. J Am Acad Audiol: 5–22 10.3766/jaaa.25.1.2 PMC5063499 24622858

[3] Cohen S, Murphy MLM, Prather AA (2019) Ten surprising facts about stressful life events and disease risk. Annu Rev Psychol: 577–597 10.1146/annurev-psych-010418-102857 PMC6996482 29949726

[4] McEwen B (2000) The neurobiology of stress: from serendipity to clinical relevance. Brain Res Brain Res Protoc: 172–189 10.1016/s0006-8993(00)02950-4 11119695

[5] Beck J (1995) Cognitive therapy: basics and beyond. Guilford, New York

[6] Moss R, Swindle R (1990) Stressful life circumstances: concepts and measures. Stress Med: 171–178

[7] Van Der Klok B (2015) The body keeps the score. Penguin, London

[8] Jones P (2019) Vox Populi. Atlantic Books, London

[9] Rowling JK (1999) Harry potter and the prisoner of Azkaban. Bloomsbury, London

[10] Baguley D (2016) Tinnitus and hyperacusis in literature, film and music. In: Baguley D, Fagelson M (eds) Tinnitus: clinical and research perspectives. Plural, San Diego, pp 1–11

[11] Tinnitus UK (2024) All about Tinnitus. https://tinnitus.org.uk/understanding-tinnitus/what-is-tinnitus

[12] Hazell J (1995) Support for a neurophysiological model of tinnitus. American Tinnitus Association. Proceedings of the Fifth International Tinnitus Seminar, Portland, pp 51–57

[13] Mazurek B, Szczepek AJ, Hebert S (2015) Stress and tinnitus. HNO: 258–265 10.1007/s00106-014-2973-7 25862619

[14] Handscomb L (2018) A systematic evaluation of the cognitive Behavioural model of Tinnitus distress. University of Nottingham, Nottingham (PhD Thesis)

[15] McKenna L et al (2014) A scientific cognitive-behavioral model of tinnitus: novel conceptualizations of tinnitus distress. Front Neurol: 196 10.3389/fneur.2014.00196 PMC4186305 25339938

[16] Goebel G, Floezinger U (2008) Pilot study to evaluate psychiatric co-morbidity in tinnitus patients with and without hyperacusis. Audiol Med: 78–84

[17] Stouffer J, Tyler R (1992) Ratings of psychological changes pre- and post-tinnitus onset. In: Arran JM, Dauman R (eds) Proceedings of the 4th international tinnitus seminar. Kugler, Amsterdam

[18] Hinton D, Chhean D, Pich V, Hofmann SG, Barlow DH (2006) Tinnitus among Cambodian refugees: relationship to PTSD severity. JOURNAL OF TRAUMATIC STRESS: 541–546 10.1002/jts.20138 16929509

[19] Fagelson MA (2007) The association between tinnitus and posttraumatic stress disorder. Am J Audiol: 107–117 10.1044/1059-0889(2007/015) 18056879

[20] Fagelson MA, Smith SL (2016) Tinnitus self-efficacy and other Tinnitus self-report variables in patients with and without post-traumatic stress disorder. Ear Hear: 541–546 10.1097/AUD.0000000000000290 26950001

[21] Fagelson MA (2016) Tinnitus in military and veteran populations. In: Baguley D, Fagelson MA (eds) Tinnitus: Clinical and Research Perspectives. Plural, San Diego, pp 75–88

[22] Carmody N, Hunter M, Eikelboom RH (2023) Help-seeker satisfaction with diagnosis and treatment of tinnitus. Int J Audiol: 1–8 10.1080/14992027.2023.2292964 38117006

[23] Husain FT et al (2018) Expectations for Tinnitus treatment and outcomes: a survey study of audiologists and patients. J Am Acad Audiol: 313–336 10.3766/jaaa.16154 29664725

[24] Dauman N et al (2017) Exploring Tinnitus-induced disablement by persistent frustration in aging individuals: a grounded theory study. Front Aging Neurosci: 272 10.3389/fnagi.2017.00272 PMC5554335 28848429

[25] Pryce H et al (2019) Tinnitus groups: a model of social support and social connectedness from peer interaction. British J Health Psychol: 913–930 10.1111/bjhp.12386 PMC6899850 31449732

[26] Pryce H, Dauman N, Burns-O’Connell G (2023) What is the burden of tinnitus? Front Psychol: 981776 10.3389/fpsyg.2022.981776 PMC9879209 36710784

[27] Marks E, Smith P, McKenna L (2019) Living with tinnitus and the health care journey: An interpretative phenomenological analysis. British J Health Psychol: 250–264 10.1111/bjhp.12351 30609202

[28] Cima RF, Crombez G, Vlaeyen JW (2011) Catastrophizing and fear of tinnitus predict quality of life in patients with chronic tinnitus. Ear Hear: 634–641 10.1097/AUD.0b013e31821106dd 21399500

[29] Wray N (2021) This is My silence: please listen. Hear J 17(16):14

[30] Probst T et al (2016) Emotional states as mediators between tinnitus loudness and tinnitus distress in daily life: Results from the “TrackYourTinnitus” application. Sci Rep: 20382 10.1038/srep20382 PMC4745045 26853815

[31] Pattyn T et al (2016) Tinnitus and anxiety disorders: a review. Hear Res: 255–265 10.1016/j.heares.2015.08.014 26342399

[32] Marciano E et al (2003) Psychiatric comorbidity in a population of outpatients affected by tinnitus. Int J Audiol: 4–9 10.3109/14992020309056079 12564510

[33] Zöger S, Svedlund J, Holgers KM (2001) Psychiatric disorders in tinnitus patients without severe hearing impairment: 24 month follow-up of patients at an audiological clinic. Audiology: 133–140 10.3109/00206090109073108 11465295

[34] Zöger S, Svedlund J, Holgers KM (2006) Relationship between tinnitus severity and psychiatric disorders. Psychosomatics: 282–288 10.1176/appi.psy.47.4.282 16844885

[35] De Ridder D et al (2021) Tinnitus and tinnitus disorder: theoretical and operational definitions (an international multidisciplinary proposal). Prog Brain Res: 1–25 10.1016/bs.pbr.2020.12.002 33637213

[36] Trevis KJ, McLachlan NM, Wilson SJ (2018) Systematic review and meta-analysis of psychological functioning in chronic tinnitus. Clin Psychol Rev: 62–86 10.1016/j.cpr.2017.12.006 29366511

[37] Wilson PH, Henry JL (1998) Tinnitus Cognitions questionnaire: development and Psychometric properties of a measure of dysfunctional Cognitions associated with Tinnitus. Int Tinnitus J: 23–30 10753381

[38] Rauschecker JP, Leaver AM, Mühlau M (2010) Tuning out the noise: limbic-auditory interactions in tinnitus. Neuron: 819–826 10.1016/j.neuron.2010.04.032 PMC2904345 20620868

[39] De Ridder D et al (2014) An integrative model of auditory phantom perception: tinnitus as a unified percept of interacting separable subnetworks. Neurosci Biobehav Rev: 16–32 10.1016/j.neubiorev.2013.03.021 23597755

[40] Husain FT (2016) Neural networks of tinnitus in humans: elucidating severity and habituation. Hear Res: 37–48 10.1016/j.heares.2015.09.010 26415997

[41] Weise C et al (2013) Acceptance of tinnitus: validation of the tinnitus acceptance questionnaire. Cogn Behav Ther: 100–115 10.1080/16506073.2013.781670 23627873

[42] Schlee W, Hølleland S, Bulla J, Simoes J, Neff P, Schoisswohl S, Woelflick S, Schecklmann M, Schiller A, Staudinger S, Probst T, Langguth B (2020) The effect of environmental stressors on Tinnitus: a prospective longitudinal study on the impact of the COVID-19 pandemic. JCM: 2756 10.3390/jcm9092756 PMC7565885 32858835

[43] Hébert S, Canlon B, Hasson D (2012) Emotional exhaustion as a predictor of tinnitus. Psychother Psychosom: 324–326 10.1159/000335043 22854311

[44] Job A, Cian C, Esquivié D, Leifflen D, Trousselard M, Charles C, Nottet JB (2004) Moderate variations of mood/emotional states related to alterations in cochlear otoacoustic emissions and tinnitus onset in young normal hearing subjects exposed to gun impulse noise. Hear Res: 31–38 10.1016/j.heares.2004.02.010 15219318

[45] Erlandsson SI, Lundin L, Dauman N (2020) The experience of Tinnitus and its interaction with unique life histories-life events, trauma and inner resources narrated by patients with Tinnitus. Front Psychiatry: 136 10.3389/fpsyt.2020.00136 PMC7093576 32256394

[46] Dauman N, Erlandsson SI (2012) Learning from tinnitus patients’ narratives—a case study in the psychodynamic approach. Int J Qual Stud Health Well-being: 1–11 10.3402/qhw.v7i0.19540 PMC3531319 23272657

[47] van Munster JJCM et al (2020) The relationship of Tinnitus distress with personality traits: a systematic review. Front Neurol: 225 10.3389/fneur.2020.00225 PMC7326028 32655464

[48] Jastreboff PJ, Hazell JW (1993) A neurophysiological approach to tinnitus: clinical implications. Br J Audiol: 7–17 10.3109/03005369309077884 8339063

[49] Hallam RS, Rachman S, Hinchcliffe R (1984) Psychological aspects of tinnitus. In: Rachman S (ed) Contributions to Medical Psychology, vol 3. Pergamon, Oxford

[50] Szczepek AJ, Mazurek B (2021) Neurobiology of Stress-Induced Tinnitus. Curr Top Behav Neurosci: 327–347 10.1007/7854_2020_215 33604876

[51] Hoskin R, Hunter MD, Woodruff PW (2014) The effect of psychological stress and expectation on auditory perception: A signal detection analysis. Br J Psychol: 524–546 10.1111/bjop.12048 25280122

[52] Sedley W et al (2016) An integrative Tinnitus model based on sensory precision. Trends Neurosci: 799–812 10.1016/j.tins.2016.10.004 PMC5152595 27871729

[53] McGilchrist I (2009) The master and his emissary: the divided brain and the making of the western world. Yale University Press, New Haven

[54] Epp B et al (2012) Increased intensity discrimination thresholds in tinnitus subjects with a normal audiogram. J Acoust Soc Am: EL196–EL201 10.1121/1.4740462 22979832

[55] Scott AR (2024) Is There Hope for People With Tinnitus. www.medscape.co.uk/viewarticle/there-hope-people-tinnitus-2024a10005ay?ecd=wnl_ret_240502_mscpmrk-GB_mostread_etid6484397&uac=410141CY&impID=6484397&sso=true. Accessed 25 May 2024 (www.medscape.co.uk)

